# An exploration of the potential utility of fetal cardiovascular MRI as an adjunct to fetal echocardiography

**DOI:** 10.1002/pd.4912

**Published:** 2016-08-31

**Authors:** David F. A. Lloyd, Joshua F. P. van Amerom, Kuberan Pushparajah, John M. Simpson, Vita Zidere, Owen Miller, Gurleen Sharland, Joanna Allsop, Matthew Fox, Maelene Lohezic, Maria Murgasova, Christina Malamateniou, Jo V. Hajnal, Mary Rutherford, Reza Razavi

**Affiliations:** ^1^ Evelina Children's HospitalLondonUK; ^2^ Division of Imaging Sciences and Biomedical EngineeringKing's College LondonLondonUK

## Abstract

**Objectives:**

Fetal cardiovascular magnetic resonance imaging (MRI) offers a potential alternative to echocardiography, although in practice, its use has been limited. We sought to explore the need for additional imaging in a tertiary fetal cardiology unit and the usefulness of standard MRI sequences.

**Methods:**

Cases where the diagnosis was not fully resolved using echocardiography were referred for MRI. Following a three‐plane localiser, fetal movement was assessed with a balanced steady‐state free precession (bSSFP) cine. Single‐shot fast spin echo and bSSFP sequences were used for diagnostic imaging.

**Results:**

Twenty‐two fetal cardiac MRIs were performed over 12 months, at mean gestation of 32 weeks (26–38 weeks). The majority of referrals were for suspected vascular abnormalities (17/22), particularly involving the aortic arch (*n* = 10) and pulmonary vessels (*n* = 4). Single‐shot fast spin echo sequences produced ‘black‐blood’ images, useful for examining the extracardiac vasculature in these cases. BSSFP sequences were more useful for intracardiac structures. Real‐time SSFP allowed for dynamic assessment of structures such as cardiac masses, with enhancement patterns also allowing for tissue characterisation in these cases.

**Conclusions:**

Fetal vascular abnormalities such as coarctation can be difficult to diagnose by using ultrasound. Fetal MRI may have an adjunctive role in the evaluation of the extracardiac vascular anatomy and tissue characterisation. © 2016 The Authors. *Prenatal Diagnosis* published by John Wiley & Sons, Ltd.

## Abstract 2

**What's Already Known About This Topic?**

- Fetal cardiac MRI offers the potential to be a safe adjunct to echocardiography; however, there is no consensus on routine fetal indications or technical protocols for cardiovascular MRI in clinical practice. Previous studies have focused mainly on its utility for intracardiac imaging.

**What Does This Study Add?**

- Abnormalities involving the extracardiac vasculature were the most common group of referrals for additional imaging in clinical practice. Fast spin echo MRI sequences offer the potential for better visualisation of these structures than ultrasound alone. Additional MRI benefits such as tissue characterisation can add further value in selected cases.

## Introduction

Conventional fetal echocardiography is the mainstay for prenatal diagnosis of congenital cardiac lesions, based on its ease of use, availability and high diagnostic accuracy.^1^, ^2^, ^3^ However, individual fetal and maternal factors can have a deleterious effect on the quality of ultrasound imaging,^4^, ^5^ and there remain inherent difficulties in diagnosing specific forms of congenital heart disease in the fetus.^3^, ^6^, ^7^, ^8^ Despite this, due to technical and safety considerations, alternatives to ultrasonographic techniques have traditionally been extremely limited.^9^

Magnetic resonance imaging (MRI) has been an established adjunct for postnatal assessment of the cardiovascular system since the 1980s,^10^ with routine studies able to deliver three‐dimensional angiography, detailed real‐time imaging, cardiac volumes and vascular flow measurements.^11^ Cardiovascular MR imaging in the fetus, however, presents several challenges not present after birth. The fetal heart is extremely small, with a total length of between 2 and 4 cm depending on gestational age,^12^ and beats with high heart rates of up to 160 beats per minute.^13^ Electrocardiographic ‘gating’, a means of improving spatiotemporal resolution, is not yet practicable in the fetus, although several non‐invasive methods are under investigation.^14^, ^15^ Unpredictable three‐dimensional fetal motion, maternal bulk motion and fetal and maternal breathing movements present additional difficulties,^16^ with no additional benefit from attempted fetal sedation.^5^

The first report of attempted fetal cardiac MRI was in 2005, using real‐time sequences with an attempt to quantify ventricular volumes.^17^ There followed a number of small case series of fetuses with normal hearts^18^, ^19^ and congenital heart disease.^20^ Whilst several subsequent reviews have attempted to develop defined protocols,^21^, ^22^ few have focused on the clinical role and utility of fetal cardiac MRI,^5^ and there is currently no consensus on routine fetal indications or technical protocols for cardiovascular MRI in clinical practice.

The increasing use of MRI for prenatal evaluation of the fetal brain^23^ and other organs^24^ offers the potential for more widespread access to the expertise and infrastructure required to deliver fetal MRI programmes. We sought to explore both the need for adjunctive fetal cardiac imaging in a tertiary fetal cardiology unit and the usefulness of standard MRI sequences in delivering additional diagnostic information.

## Aims

Over a one‐year period, fetal MRI was requested on clinical grounds by the fetal cardiology service, reserved for any difficult fetal cases where the diagnosis was in doubt or could benefit from additional imaging. We present an analysis of the pattern of referrals we subsequently observed and the diagnostic utility of standard MRI sequences in this context.

## Methods

Referrals were based on the judgement of the attending fetal cardiologists (JS, OM, VZ and GS). All examinations took place between June 2014 and May 2015. Pre‐existing ethical approval was in place for a long‐standing project examining brain growth in fetuses with CHD, which also permitted the use of cardiac sequences (REC: 07/H0707/105). All participants provided written consent and received concomitant fetal brain imaging. The timing of the MRI was determined by the clinical teams, taking into account the gestational age at diagnosis, the potential impact of new information on fetal counselling and management, and the timeframe for optimal imaging quality (generally later gestation, when the fetus is larger with fewer major translational movements). A paediatric cardiologist with expertise in cardiac MR was present at every study (RR or KP). Each mother's tympanic temperature was taken before and after the MRI or if they became uncomfortable during the study. Conservative limits of a rise in temperature of ≥1 °C and/or an absolute estimated temperature in the fetus of greater than 38 °C (i.e. maternal temperature +0.5 °C)^17^ were set; breach of either would lead to the study being discontinued.

A large field of view (covering the whole fetus) bSSFP cine (real‐time) scan with low spatial resolution but higher temporal resolution (303 ms) was used following the initial localiser sequence to demonstrate the degree of gross fetal movement.^25^ Diagnostic sequences were generally limited to half‐Fourier acquisition single‐shot fast spin‐echo (‘SSFSE’) and balanced steady‐state free precession (‘bSSFP’) gradient echo sequences, as these sequences were felt to offer reasonable signal contrast and spatiotemporal resolution within established limits of radiofrequency power deposition^26^ and acoustic noise^27^. These were either multi‐slice imaging to provide whole heart coverage using parallel overlapping slices or single‐slice imaging in targeted scan planes where dynamic information was of interest (real‐time; Table 1). If needed, further shortened localiser sequences were also used to account for fetal movement. Sequences were repeated as needed to a maximum study time of 60 min.

**Table 1:** Imaging parameters

Sequence	Multi‐slice SSFSE	Multi‐slice bSSFP	Single‐slice bSSFP
TR/TE (ms)	15 000/100	4.3/2.1	3.9/1.9
Flip angle (degrees)	90	90	60
Field of view (mm)	350 × 350	350 × 350	400 × 300
In plane resolution (mm)	1.4 × 1.4	1.5 × 2.3	1.8 × 2.3
Slice thickness (mm)	2.5	5	5
Order	16 interleaved/TR	Linear slices	Dynamic single slice
SENSE factor	2	1	2
Partial‐Fourier factor	5/8	1	5/8
Single‐slice duration (ms)	468	647	159

SSFSE, single shot fast spin echo; bSSFP, balanced steady‐state free precession; TR, repetition time; TE, echo time; SENSE, sensitivity encoding.

## Results

Twenty‐two fetal cardiac MRI examinations were performed over the referral period. The average gestational age at diagnosis 23^+4^ weeks (median 23^+3^ weeks, range 15^+4^–34^+0^ weeks) and the average age at MRI was 31^+3^ weeks (median 33^+2^ weeks, range 22^+2^–38^+1^ weeks). Two twin pregnancies were included, each with one healthy and one affected twin.

The most common indications were for possible coarctation of the aorta in six patients, other arch abnormalities in four patients (including aortic interruption in two patients, right aortic arch and aberrant left subclavian artery) and assessment of pulmonary vessels in four patients (Table 2). In total, referrals for clarification of the extracardiac cardiovascular anatomy comprised 77% of all referrals (17/22 cases). No examinations were abandoned early for maternal claustrophobia. One patient experienced a rise in temperature to 38.3 °C towards the end of the study, which was then discontinued.

**Table 2:** Referral indications

Indication	**N**	MRI successful	No cardiac data obtained	No postnatal data
Possible coarctation	6	5	1^a^	—
Aortic arch anatomy	4	3	1^a^	—
Pulmonary vasculature	4	3	1^b^	—
Other vascular	3	3	—	1 (TOP 35w)
Cardiac mass	3	3	—	1 (TOP 32w)
Cardiac diverticulum	2	2	—	—
TOTAL (%)	22 (100%)	19 (86%)	3 (14%)	2 (9%)

TOP, termination of pregnancy.

a Excessive fetal movement.

b Unable to visualise pulmonary veins due to lung compression from large hernia.

Three examinations failed to provide any useful cardiovascular information. In two cases, this was due to excessive fetal movement (at 29 and 33 weeks). In one case, assessment of the pulmonary veins in a 26‐week fetus was impossible due to severe lung compression from a large diaphragmatic hernia; however, this important prognostic information was used to help counsel the parents prior to the demise of the fetus at 29 weeks. No postnatal data were available in two cases (one intrauterine death at 32 weeks and one medical termination at 35 weeks). Autopsy was not performed in either case. In all remaining patients, the MRI diagnosis was confirmed postnatally. A comprehensive summary of all studies performed is shown in Table 3.

**Table 3:** Summary table of all fetuses referred for fetal cardiac MRI

**GA**										
*n*	W	D	Fetal diagnosis (USA)	MRI indication	Co‐morbidity	MRI findings	Additional MRI benefits	Postnatal diagnosis	Outcome	Age
1	29	2	AVSD Ventricular asymmetry	Evidence of CoA?	Cerebral ventriculomegaly	Abandoned: excessive fetal movement	Concomitant MRI brain	Borderline left heart Coarctation of the aorta (echocardiography and surgery)	Hybrid procedure	5 days
2	32	2	Possible coarctation	Evidence of CoA?	None	Hypoplasia of the aortic isthmus ‘Posterior shelf’ (Figure 1) Ventricular asymmetry LV < RV High risk of coarctation	—	Coarctation of the aorta (echocardiography and surgery)	Coarctation repair	3 days
3	35	3	Hypoplastic left heart syndrome	Pulmonary venous anatomy?	None	Large, tortuous, solitary pulmonary veins bilaterally (Figure 3)	—	As per prenatal imaging (postnatal MRI and surgery)	Hybrid procedure	3 days
4	33	3	?Right ventricular aneurysm Small pericardial effusion	Ventricular aneurysm/ diverticulum?	None	Normal cardiac connections No aneurysm seen	—	Structurally normal heart (echocardiography)	Outpatient review	—
5	34	0	Hypoplastic left heart syndrome Restrictive atrial septum	Pulmonary venous anatomy?	None	Dilated pulmonary veins with normal anatomy	No pulmonary lymphangectasia	As per prenatal imaging (echocardiography and surgery)	Hybrid procedure	4 days
6	34	0	Muscular VSD Possible coarctation	Evidence of CoA?	None	Mild ventricular asymmetry Normal arch and isthmus Low suspicion of coarctation	—	Structurally normal heart (echocardiography)	Outpatient review	—
7	33	2	Left atrial mass	Nature of cardiac mass?	Possible tuberous sclerosis	Left atrial mass: atrial myxoma, haemangioma or rhabdomyoma (Figure 7)	Tissue characterisation	Left atrial rhabdomyoma (histology)	Surgical resection	—
8	38	1	RV apex tumour	Nature of cardiac mass?	None	Solitary RV rhabdomyoma (Figure 8)	Tissue characterisation Concomitant MRI brain	As per prenatal imaging (echocardiography)	Outpatient review	—
9	33	5	Mal‐aligned VSD Interrupted aortic arch	Aortic arch anatomy?	22q11 microdeletion (postnatal)	Type B interruption Subclavian in continuity with descending Ao	—	As per prenatal imaging (echocardiography and surgery)	Surgical repair	2 days
10	33	6	Common arterial trunk ?Interruption of aortic arch	Aortic arch anatomy?	22q11 microdeletion Left hydronephrosis	Common arterial trunk with arterial duct No interruption	—	As per prenatal imaging (postnatal MRI and surgery)	Surgical repair	9 days
11	28	2	Twin 1: normal Twin 2: multiple cardiac masses	Nature of cardiac masses?	DCDA twins	Twin 1: normal Twin 2: three intramyocardial tumours suggestive of rhabdomyomas on T2 weighting	Tissue characterisation MRI brain: multiple rhabdomyomas Twin 1: normal heart	Selective feticide: no post‐mortem Maternal germ‐line mosaicism for tuberous sclerosis	Twin 1 well, twin 2: selective feticide	32/40
12	32	1	?Coarctation of the aorta Bilateral SVCs	Evidence of CoA?	None	Hypoplasia of the aortic isthmus Long gap LCCA–LSA High‐risk neonatal coarctation (Figure 4)	—	Mild coarctation of the aorta only (Figure 5) (postnatal MRI)	No intervention to date	29 days
13	29	6	Dysplastic pulmonary valve Severe pulmonary regurgitation Maternal obesity	Pulmonary artery anatomy?	None	Confluent right and left PAs	—	As per prenatal imaging (postnatal CT)	Outpatient review	—
14	33	4	Right‐sided aortic arch Left‐sided arterial duct ALSA	Aortic arch anatomy?	None	Inadequate views (fetal motion)	—	Right‐sided aortic arch ALSA (echocardiography)	Outpatient review	—
15	33	5	Small aortic arch ?Coarctation of the aorta	Evidence of CoA?	None	Moderate transverse arch hypoplasia High‐risk coarctation	—	Coarctation of the aorta (echocardiography and surgery)	Surgical repair	6 days
16	36	5	Twin 1: R arch, L duct, ALSA Twin 2: normal	Aortic arch anatomy?	None	Twin 1: R arch, L duct, ALSA Twin 2: normal	No airway compression	As per prenatal imaging (postnatal MRI)	Outpatient review	—
17	33	1	Cardiomegaly, RV>LV Dilated great vessels Normal aortic isthmus	Other vascular: exclude large AVMs	None	Large RV, PA and Ao Normal connections No AVMs	—	As per antenatal imaging (echocardiography)	Outpatient review	—
18	26	4	Diaphragmatic hernia	Pulmonary venous anatomy?	Diaphragmatic hernia	Not able to visualise pulmonary veins due to lung compression	Large diaphragmatic hernia with pulmonary hypoplasia	Normal pulmonary veins Absent diaphragm bilaterally (post‐mortem)	Medical termination	29/40
19	30	3	Possible RV diverticulum	Ventricular aneurysm/ diverticulum?	SCD	Wide‐mouthed contractile diverticulum at base of right ventricle, 10 × 12 mm (Figure 6)	—	As per prenatal imaging (echocardiography)	Outpatient review	—
20	28	0	Abnormal arterial duct	Other vascular: arterial duct	None	Leftward rotation of cardiac axis with tortuous arterial duct Normal vascular anatomy	—	Structurally normal heart (echocardiography)	Outpatient review	
21	22	4	Bilateral SVCs, apex to right ?Other vascular abnormality	Other vascular	Jacobsen syndrome	Rightward rotation of cardiac axis Bilateral SVCs Normal vascular anatomy	—	Medical termination Genetics: Jacobsen syndrome (no post‐mortem)	Medical termination	35/40
22	22	2	Bilateral SVCs ?Coarctation of the aorta	Evidence of CoA?	Deficient cerebellar vermis	Dominant RV, bilateral SVCs, hypoplastic aortic arch, high probability of coarctation	Deficient cerebellar vermis diagnosed	Coarctation of the aorta (echocardiography)	Premature Died 1 day of age (atrial arrhythmia)	33/40

AVSD, atrioventricular septal defect; CoA, coarctation of the aorta; LV, left ventricle, RV, right ventricle; VSD, ventricular septal defect; LCCA, left common carotid artery; LSA, left subclavian artery; PA, pulmonary artery; ALSA, aberrant left subclavian artery; LPA, left pulmonary artery; DCSA, doubly committed sub‐arterial; AVM, arteriovenous malformation; Ao, aorta; CHAOS, congenital high airway obstruction syndrome; SCD, sickle cell disease.

### MRI sequences

**Figure 1:**
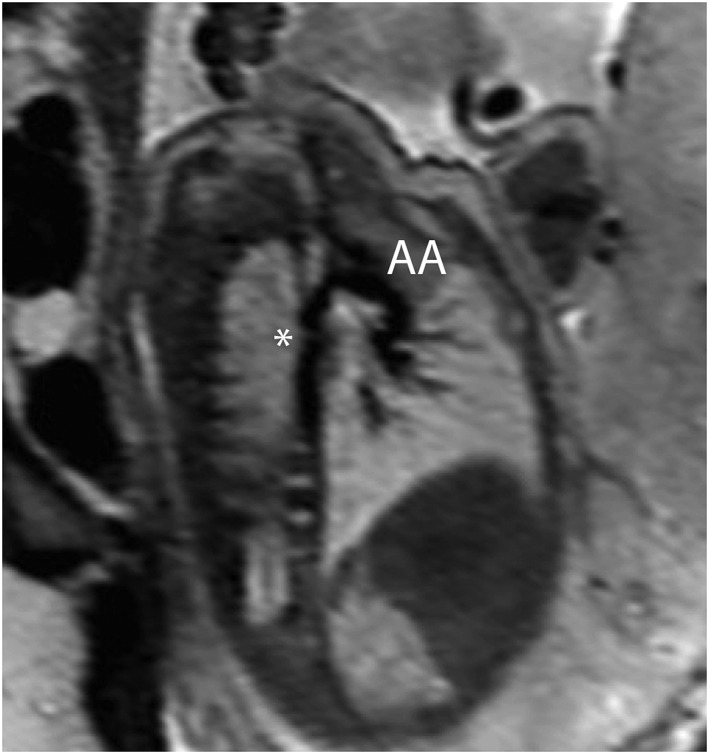
Single‐shot fast spin‐echo (SSFSE) black‐blood image of the aorta in a 32‐week fetus with coarctation of the aorta, confirmed postnatally. A characteristic indentation in the region of the aortic isthmus (a ‘posterior shelf’) is clearly visualised (*). AA, aortic arch

**Figure 2:**
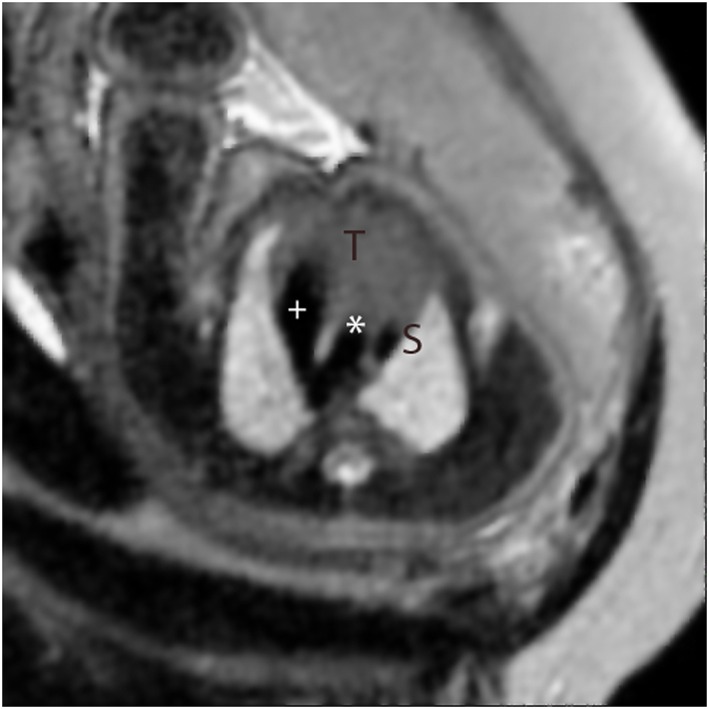
Single‐shot fast spin‐echo (SSFSE) black‐blood image in a high transverse orientation in a normal fetus. This corresponds to the standard ‘three‐vessel view’ in fetal echocardiography showing the V‐shaped connection between arterial duct (+) and the aorta (*) adjacent to the superior caval vein (S). The thymus gland is seen anteriorly (T)

**Figure 3:**
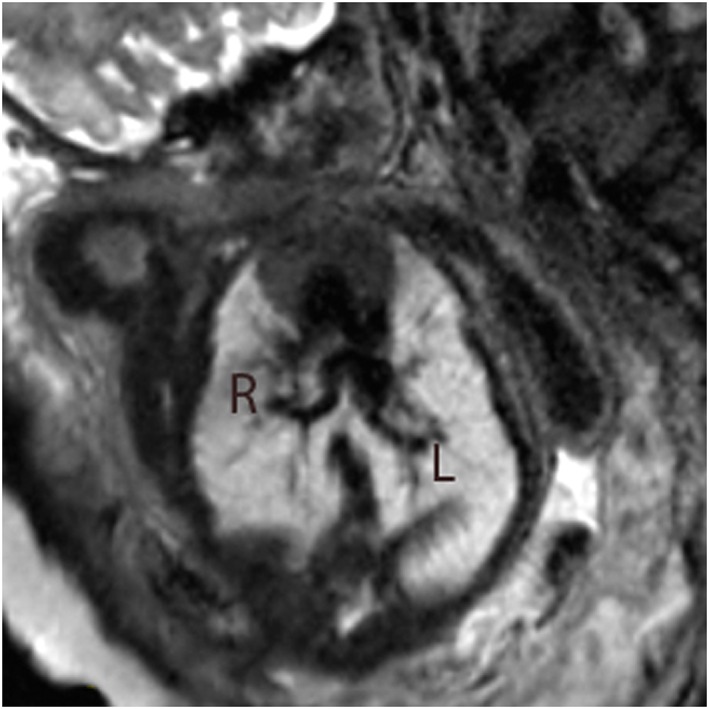
Single‐shot fast spin‐echo (SSFSE) black‐blood image in a 35‐week fetus with hypoplastic left heart syndrome with highly unusual pulmonary venous drainage. Tortuous, solitary pulmonary veins are seen bilaterally draining to confluence connected directly to the left atrium. R, right pulmonary vein; L, left pulmonary vein

**Figure 4:**
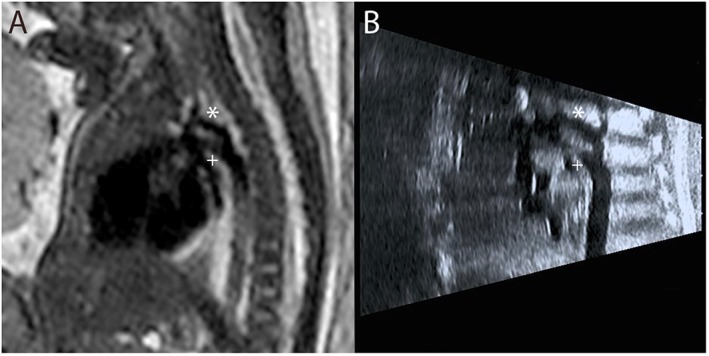
Single‐shot fast spin‐echo (SSFSE) black‐blood image in a 32‐week fetus with suspected coarctation of the aorta. The hypoplastic aortic arch (*) is visualised superior to the dominant ductal arch (+) on fetal MRI (A), confirming prenatal ultrasound findings (B)

**Figure 5:**
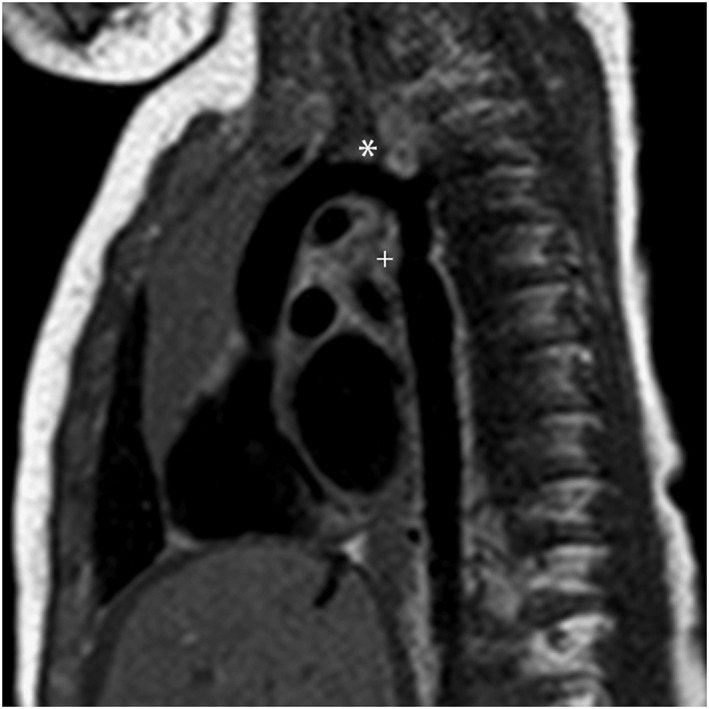
Postnatal MRI of the same patient at 5 months of age. No postnatal interventions have been performed. Despite prenatal findings, there is only mild coarctation, with isthmal hypoplasia and a posterior shelf at the point of insertion of the ductal ligament (+) (a vestige of the fetal arterial duct). The proximal aortic arch is within normal limits (*)

Single‐shot fast spin echo sequences produced T2‐weighted images with black‐blood‐like contrast with excellent contrast between the vasculature and higher signal intensity of the thymus, lung and myocardium. These sequences were the most useful for assessment of extra‐cardiac vascular anatomy as these structures are less mobile throughout the cardiac cycle (Figures 1, 2, 3, 4). They were, however, still prone to artefact from gross fetal movement. It is important to note that three‐dimensional interrogation of multi‐slice sequences was not possible, as even small fetal movements produced non‐contiguous anatomy between slices (see the *Future directions* section). An example of a standard postnatal ‘black‐blood’ sequence is shown in Figure 5.

**Figure 6:**
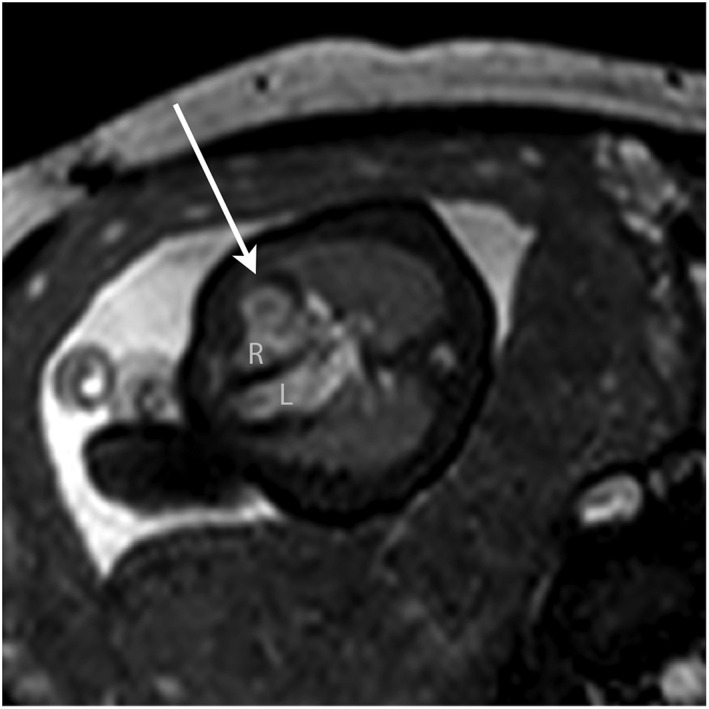
Still from a bSSFP cine (real‐time) sequence in a 30‐week fetus with a large right ventricular diverticulum (arrowed). R, right ventricle; L, left ventricle

**Figure 7:**
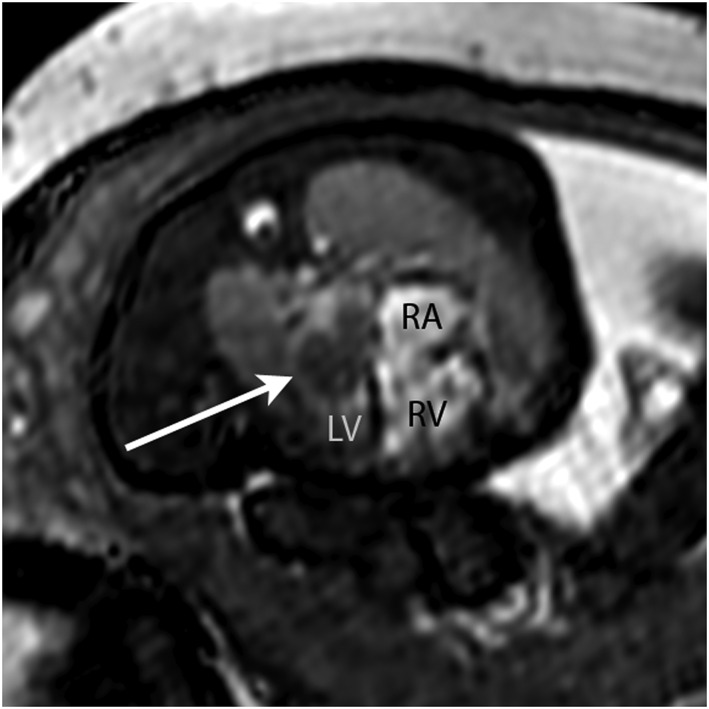
Still from a bSSFP cine (real‐time) sequence in a 33‐week fetus with a large left atrial mass (arrowed). The MRI differential diagnosis included myxoma, haemangioma or rhabdomyoma. The latter was confirmed postnatally. RA, right atrium; RA, right ventricle; LV, left ventricle

**Figure 8:**
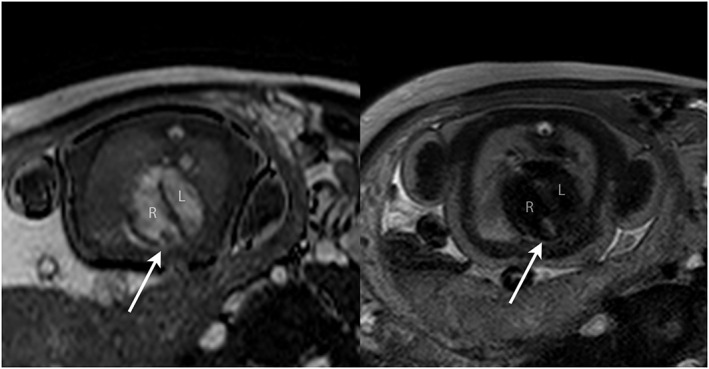
Balanced SSFP bright blood image (A) and single‐shot fast spin‐echo (SSFSE) black‐blood image (B) in a 38‐week fetus with an RV mass (arrowed). Based on the size, position and tissue signal on multiple sequences, a solitary ventricular rhabdomyoma was suspected; no other masses were seen including on concomitant fetal brain MRI. All findings were confirmed postnatally. R, right ventricle; L, left ventricle

Balanced SSFP images produced bright blood images with good contrast between the blood pool and surrounding tissue, particularly intracardiac structures (Figures 6, 7, 8); however, they were more susceptible to motion and/or flow artefacts. Real‐time bSSFP allowed for dynamic imaging of the beating fetal heart and was the most reliable sequence for assessment of moving structures, i.e. aneurysms and diverticulums (Figure 6). Differences in enhancement patterns between sequences allowed for tissue characterisation of intracardiac masses by using established MRI techniques.^28^

## Discussion

We instituted an open referral process for fetal MRI in a tertiary UK fetal cardiac centre. Whilst previous explorations of the clinical utility of fetal cardiac MRI have focused predominantly on intracardiac anatomy,^5^ the most common indication in our series was clarification of the extracardiac vascular anatomy, in keeping with established evidence that abnormalities involving these vessels, such as the coarctation of the aorta^8^, ^29^, ^30^ and anomalies of the pulmonary veins,^6^, ^7^ are amongst the most challenging to define using ultrasound alone. Novel 3D and 4D echocardiographic techniques, such as spatiotemporal image correlation, have shown some potential for adjunctive imaging and can be visualised in three dimensions, but are still subject to many of the same limitations as conventional ultrasound.^12^ Promisingly, however, and in keeping with animal models,^31^ our initial data suggest a promising strength of fetal MRI in vascular imaging, particularly using SSFSE sequences. To date, the only other report examining the clinical utility of fetal cardiac MRI used bSSFP sequences only, at a similar median gestational age to our series, interpreted blindly in 40 fetuses with CHD. Whilst this showed reasonable sensitivity for detection of abnormal intracardiac anatomy, the authors concede that MRI added no new diagnostic information when compared to ultrasound. In the same study, however, 15 fetuses were classified postnatally as having an abnormal aortic arch, of which five had been missed on ultrasound, six were missed on MRI and four were detected on both.^5^ Unlike in our study, no SSFSE sequences were used for vascular imaging.

The single most frequent single referral indication in our series was for suspected coarctation of the aorta, a notoriously challenging condition to predict prenatally due to both the technical difficulty of imaging this region and the obligatory patency of the arterial duct in fetal life, the postnatal closure of which can only then reveal life‐threatening aortic constriction.^7^, ^8^, ^30^ Alongside extracardiac and genetic associations, a number of anatomical fetal risk factors for the condition have been suggested, such as the absolute and relative diameter of aortic isthmus, the presence of a posterior shelf, ventricular/great artery asymmetry and the persistence of a left‐sided superior caval vein.^8^ In fetal MRI, imaging quality is unaffected by artefact from the fetal spine and ribcage, offering the potential for more accurate visualisation of these subtle anatomical factors. It is well established, however, that even a detailed anatomical assessment is not sufficient to accurately predict postnatal coarctation; for example, one of our cases with clearly visualised hypoplasia of the aortic arch before birth has gone on to develop only a mild form of coarctation, which has yet to require intervention (Figures 4 and 5). Indeed, it is likely that coarctation has a particularly complex aetiology, not yet fully understood, related to intrinsic myocardial abnormalities and/or a reduction in flow through the left heart. This has only recently started to be explored by using functional ultrasound techniques, rather than relying on anatomical findings alone.^32^ The addition of more advanced MRI techniques in the future may help to define a more robust method of stratifying fetuses with suspected coarctation, providing a more detailed physiological assessment of affected fetuses (see the *Future directions* section).

In addition to vascular imaging, in three cases the combined use of SSFSE sequences provided the unique ability to characterise cardiac tumours before birth, a potential use of prenatal MRI with a high prognostic impact.^33^ Multi‐slice and real‐time bSSFP sequences were most useful for intracardiac imaging, particularly for demonstrating the anatomy of gross lesions such as cardiac masses and diverticulums.

Finally, it should also be noted that several of the MRI studies reported in our series concomitantly provided important complementary imaging that could directly or indirectly impact the cardiac prognosis, for example describing the devastating extent of pulmonary compression from a very large diaphragmatic hernia in a fetus that did not survive to term. Fetal MRI can also be used to assess lung parenchymal lesions and airway compromise in CHD^34^ and has been used to demonstrate the presence of pulmonary lymphangectasia in a fetus with hypoplastic left heart syndrome, an important prognostic finding.^35^ MRI of the fetal brain may provide crucial information to clinicians and parents regarding medium‐term and long‐term prognoses, which may have a profound effect on both prenatal and postnatal management independent of the underlying cardiac diagnosis.^23^, ^36^, ^37^

### Future directions

As we observed, even small fetal movements can disrupt the spatial relationship of multi‐slice sequences, making post‐hoc analysis extremely challenging. The application of novel motion‐corrected slice‐volume registration algorithms to multiple MRI image stacks has been used to generate accurate 3D volumes of the fetal thorax.^38^ These exciting techniques have the potential to further enhance the capability of fetal MRI for vascular imaging, both by improving image quality and reducing the gestation age at which useful MR data can be obtained, and are currently under investigation by our unit.^39^

Other advanced MRI techniques can offer novel means of evaluation of physiological parameters of the fetal heart and circulation. The application of retrospective ‘metric‐optimised’ gating to phase contrast sequences can be used to quantify vascular flows and is currently being explored in a wide variety of settings.^40^, ^41^, ^42^, ^43^ Related technology allows for the retrospective gating of single slice real‐time sequences to dramatically improve the spatiotemporal resolution for cardiac imaging.^44^ The potential extension of these real‐time sequences to whole‐heart coverage allows for the possibility of performing comprehensive volumetric measurements throughout the cardiac cycle, providing estimates of chamber volumes and ventricular function.

These innovations will not only help to bring prenatal cardiovascular MRI closer to its postnatal counterpart but could also offer the potential to enhance our fundamental understanding of the nature and aetiology of congenital heart defects – including challenging prenatal diagnoses such as coarctation of the aorta – and their interactions with other organ systems such as the placenta^45^ and the developing fetal brain.^37^ The incorporation of all of the above techniques is the subject of ongoing research within our departments.

## Conclusion

Abnormalities of the fetal vasculature such as coarctation of the aorta can be challenging to diagnose with ultrasound, even in experienced hands. Standard sequence MRI may have an adjunctive role in the assessment of the extracardiac vascular anatomy and tissue characterisation. Developments such as motion‐corrected slice‐volume registration and metric‐optimised gated phase contrast flow quantification and real‐time techniques, alongside increasingly accessible infrastructure, may further develop the role of MRI as an adjunct to echocardiography in the investigation and diagnosis of congenital heart disease in the fetus.

## Limitations

We have described 22 cases that were selected for additional imaging from a single institution. Due to the increased likelihood of image corruption from uncontrolled fetal movement, we tended to perform additional imaging in the third trimester. Whilst our median gestational age of 33 weeks is comparable to other published series, this does represent a current limitation of the modality. As imaging was performed for clinical evaluation, the acquisition and interpretation of MRI images was not blinded to previous assessments. In two fetuses that did not survive to term, no autopsy information was available due to lack of parental consent.

**What's Already Known About This Topic?**

- Fetal cardiac MRI offers the potential to be a safe adjunct to echocardiography; however, there is no consensus on routine fetal indications or technical protocols for cardiovascular MRI in clinical practice. Previous studies have focused mainly on its utility for intracardiac imaging.

**What Does This Study Add?**

- Abnormalities involving the extracardiac vasculature were the most common group of referrals for additional imaging in clinical practice. Fast spin echo MRI sequences offer the potential for better visualisation of these structures than ultrasound alone. Additional MRI benefits such as tissue characterisation can add further value in selected cases.
